# Vitamins in Cereals: A Critical Review of Content, Health Effects, Processing Losses, Bioaccessibility, Fortification, and Biofortification Strategies for Their Improvement

**DOI:** 10.3389/fnut.2021.586815

**Published:** 2021-06-16

**Authors:** Monika Garg, Anjali Sharma, Shreya Vats, Vandita Tiwari, Anita Kumari, Vibhu Mishra, Meena Krishania

**Affiliations:** ^1^Agri-Biotechnology, National Agri-Food Biotechnology Institute (NABI), Mohali, India; ^2^Food Engineering and Nutrition, Center of Innovative and Applied Bioprocessing, Mohali, India

**Keywords:** vitamins, cereals, wheatgrass, cooking losses, fortification, biofortication

## Abstract

Around the world, cereals are stapled foods and good sources of vitamins A, B, and E. As cereals are inexpensive and consumed in large quantities, attempts are being made to enrich cereals using fortification and biofortification in order to address vitamin deficiency disorders in a vulnerable population. The processing and cooking of cereals significantly affect vitamin content. Depending on grain structure, milling can substantially reduce vitamin content, while cooking methods can significantly impact vitamin retention and bioaccessibility. Pressure cooking has been reported to result in large vitamin losses, whereas minimal vitamin loss was observed following boiling. The fortification of cereal flour with vitamins B1, B2, B3, and B9, which are commonly deficient, has been recommended; and in addition, region-specific fortification using either synthetic or biological vitamins has been suggested. Biofortification is a relatively new concept and has been explored as a method to generate vitamin-rich crops. Once developed, biofortified crops can be utilized for several years. A recent cereal biofortification success story is the enrichment of maize with provitamin A carotenoids.

## Introduction

Cereals are essential foods around the world, with wheat, rice, maize, oat, barley, millets, sorghum, rye, triticale, and fonio representing the most commonly grown grains. Cereals, such as wheat, rice, and maize, are cultivated on vast scales and contribute to the global food supply (1). Cereal grains are the edible seeds of grasses and include the germ, endosperm, and bran. These staple foods can be stored for an extended period without affecting their nutritional value. A significant source of macro- and micro-nutrients, cereal grains contain carbohydrates, proteins, dietary fibers, vitamins, and minerals (2). The proportion of nutrients varies between different cereal crops and is dependent on processing and cooking methods. In comparison to the endosperm, there is a higher amount of nutrients within the seed coating, and therefore, the use of grain milling to generate refined flour reduces the nutrient content (3).

**Figure 1:**
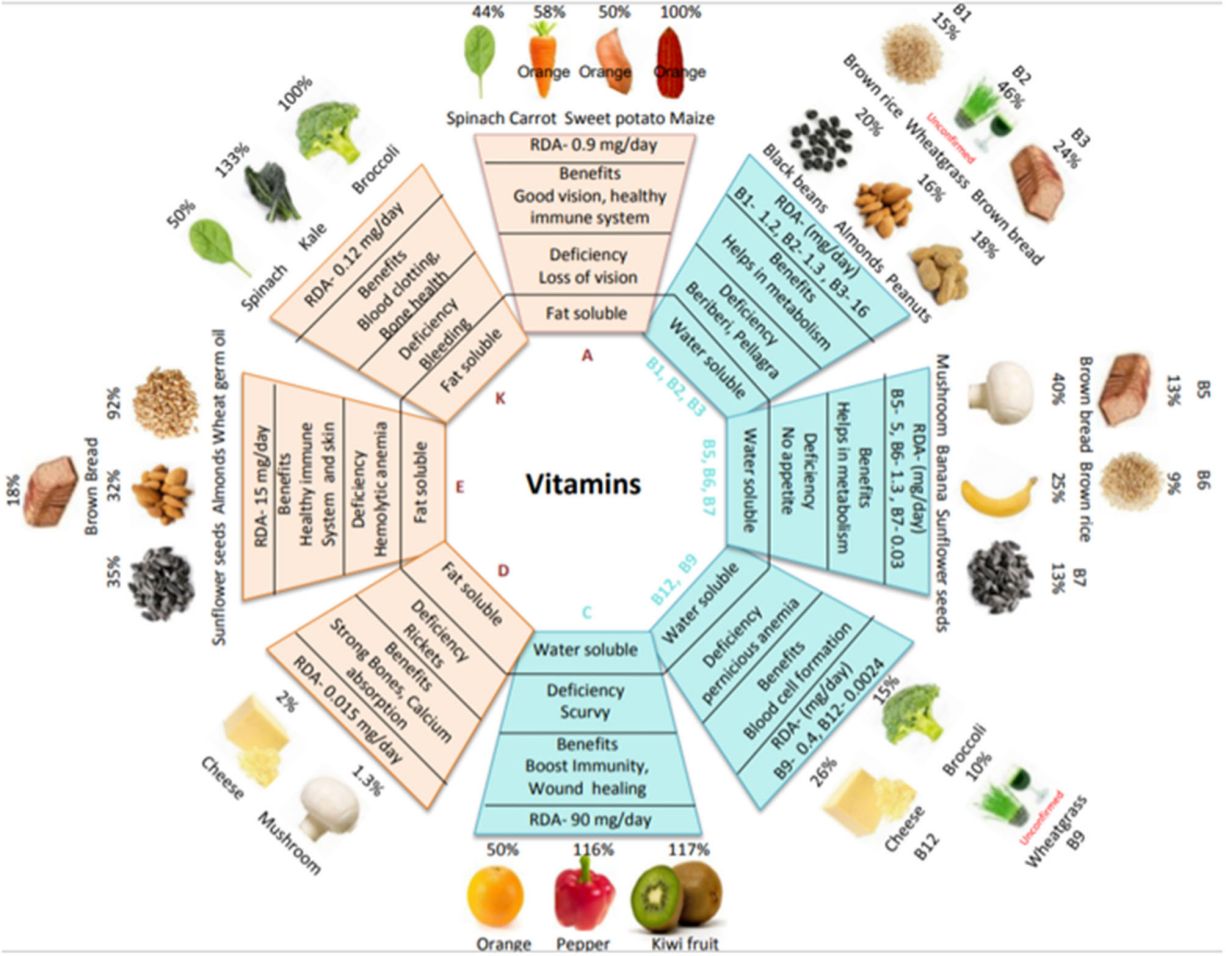
Vitamin types, food sources, benefits, and deficiencies. The diagram shows the RDA requirement of each vitamin, a variety of plant-based food sources to ensure an adequate supply of vitamins, primary functions, and deficiency disease symptoms. The %RDA for various food sources is based on adult males and is calculated using a common serving size: raw carrot, cooked spinach, canned mashed sweet potato, biofortified orange maize, boiled black beans, white mushrooms, banana, cooked broccoli, orange, raw red pepper, and kiwifruit per 100 g; dry roasted almonds, peanuts, sunflower seeds and cheese per 20 g; wheat germ oil per 5 g; and wheat grass powder per 3.5 g. Adapted from [(5–7), USDA and Food Data Central].

Vitamins are essential micronutrients for growth, metabolism, reproduction, and general well-being. Based on their solubility, vitamins are classified into two groups: fat-soluble (A, D, E, and K) and water-soluble (B and C) (4). The dietary intake of vitamins is crucial because, except for vitamins D and B1, the human body cannot synthesize them. Vitamin deficiency can lead to various disorders; however, as vitamins are present in staple foods, the consumption of vitamin-containing foods can alleviate such disorders. Figure 1 shows the main food sources of vitamins, the percent recommended daily allowance (%RDA) provided by each food source and the %RDA provided by cereals along with the associated health benefits of each vitamin (more details are provided in Supplementary Table 1). Values have been calculated from dietary reference intake recommendations for male healthy adults (19+ years) according to the United States Food and Drug Administration (FDA). Foods rich in provitamin A carotenoids include orange-colored fruits (papaya and mango) and vegetables (carrot and sweet potato); vitamins B, C, and K are found in kiwis and bananas and in vegetables such as spinach, kale, broccoli, mushrooms, and peppers; while foods such as nuts (almonds and peanuts) and cereal germ (wheat and brown rice) provide rich sources of vitamin E. Cereals are a good source of vitamins A, B, and E but are less abundant in other vitamins. The RDA for vitamin K can be easily met by consuming vegetables (e.g., spinach, kale, and broccoli). The RDA for vitamin D is difficult to meet from food sources alone; however, sunlight is a good source. In healthy adults, deficiencies in vitamins B5, B6, B7, C, and K are uncommon. However, in the vulnerable population (e.g., the elderly, infants, children, and pregnant/lactating women), the RDA for vitamins A, B2, B3, B6, B9, B12, C, D, E, and K increases. Diseases of the liver reduce the absorption of vitamins A, D, E, and K from the intestine; likewise, ileac diseases reduce vitamin B12 absorption and renal diseases reduce vitamin D absorption (8).

In line with changes in lifestyles, the consumption of cereals is rapidly increasing. Cereals are the main ingredients in many food products, e.g., porridge, breakfast cereals, raised bread, flatbread, biscuits, cereal-based beverages, and seedling juices. Before consumption, cereal grains are cooked and processed using heat treatment methods, such as steaming, blanching, boiling, and microwaving. The cooking or heat treatment method can significantly impact vitamin content; therefore, it is necessary to establish the nutritional information of the processed foods and any losses resulting from processing or cooking (9). Furthermore, the bioaccessibility of vitamins to the human body is essential for estimating their health benefits; cereals with high nutrient losses following cooking might have better bioaccessibility, and vice versa (10).

Recently, cereal seedling juice, and particularly wheatgrass juice, has received substantial attention as it is high in nutrients. Wheatgrass is known to be a potent medicine in the treatment of cardiovascular diseases (11), ulcerative colitis (12), thalassemia (13), hepatic injury (14), diabetes (15), and cancer (16); and in addition, it may also provide improved immunity (17). However, there are limited studies on their vitamin content, which poses a challenge for widespread utilization.

To address micronutrient malnutrition, attempts have been made to increase the vitamin content of cereals using fortification (the addition of vitamins) and biofortification (crop modification to increase production). The fortification of cereals has been recommended by the United States FDA, as well as several other countries. Guidelines for the fortification of refined flour with vitamins have been published. Biofortification is encouraged over the fortification, as biofortified foods provide more bio-available nutrients to the human body and carry a lower risk of overdose than fortified foods (18). In comparison to fortification, biofortification is more sustainable because, once developed, biofortified crops can be utilized for several years, whereas the fortification of foods has to be continued (19).

Although the information on vitamins is available for different crops, compiled information on cereals is not available. Therefore, in this review, we compiled the latest available information on vitamins in cereals. A literature search was conducted using electronic databases: Pubmed/Medline, Scopus, Mendeley, and Google Scholar with the objective to compile up-to-date information on variations in the vitamin content of major cereal grains and cereal grasses, %RDA values, the effects on human health, processing and cooking losses, bioaccessibility, and fortification and biofortification strategies.

## Vitamins in Whole Grain Cereals

Cereals constitute an important part of our diet. The most important cereals in terms of consumption are wheat, rice, and corn (20). These cereals contain many vitamins and minerals that are essential for human health. Whole grain cereals are rich in vitamins A, B1, B2, B3, B5, B6, B9, E, and K, although they are not good sources of vitamins B12, C, and D [(21); Table 1]. Cereals are processed in several ways to produce a variety of foods, and the method of processing can significantly impact vitamin content. Cereals can be consumed whole or milled; milling to produce flour can result in a substantial vitamin content reduction, depending on its location in the grain (Table 2). The vitamin content present in the final product consumed plays a vital role in the functioning of the human body.

**Table 1:** Vitamin content (mg/100 g) in whole grains and their refined forms.

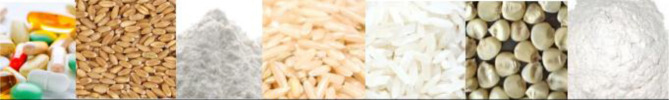									
**Vitamin**	Wheat^*^ (whole)	**Wheat (milled)**	**Loses (%)**	**Rice^b^(Whole)**	**Rice^b^(milled)**	**Loses (%)**	**Corn^a^ (whole)**	Corn^*^^a^ (Degermed)	**Loses (%)**
B 1	0.38	0.12	68	0.34	0.07	79	0.39	0.1092	72
B 2	0.11	0.04	64	0.09	0.03	67	0.2	0.088	56
B 3	4.38	1.25	71	4.62	1.60	65	3.6	2.16	40
B 5	0.95	0.43	55	0.92	0.45	51	0.42	0.050	88
B 6	0.36	0.07	80	1.3	0.75	42	0.62	0.217	65
B 9	0.56	0.185	66	0.03	0.01	67	0.26	0.085	67
E^**^	1.01/4.05	0.06/1.8	94/55	1.7/NA	0.14/NA	92	2.4/7.9	0.12/2.0	95/75
K	0.002	0.0003	85	0.0006	0.0001	83	0.0003	NA	NA

*Source- USDA Food data central;*

a,b *(21);*

* *White;*

** *α Tocopherol/total tocols; NA, not available from reliable source*.

**Table 2:** Vitamin content (mg/100 g) and % RDA of cooked whole and refined cereals.

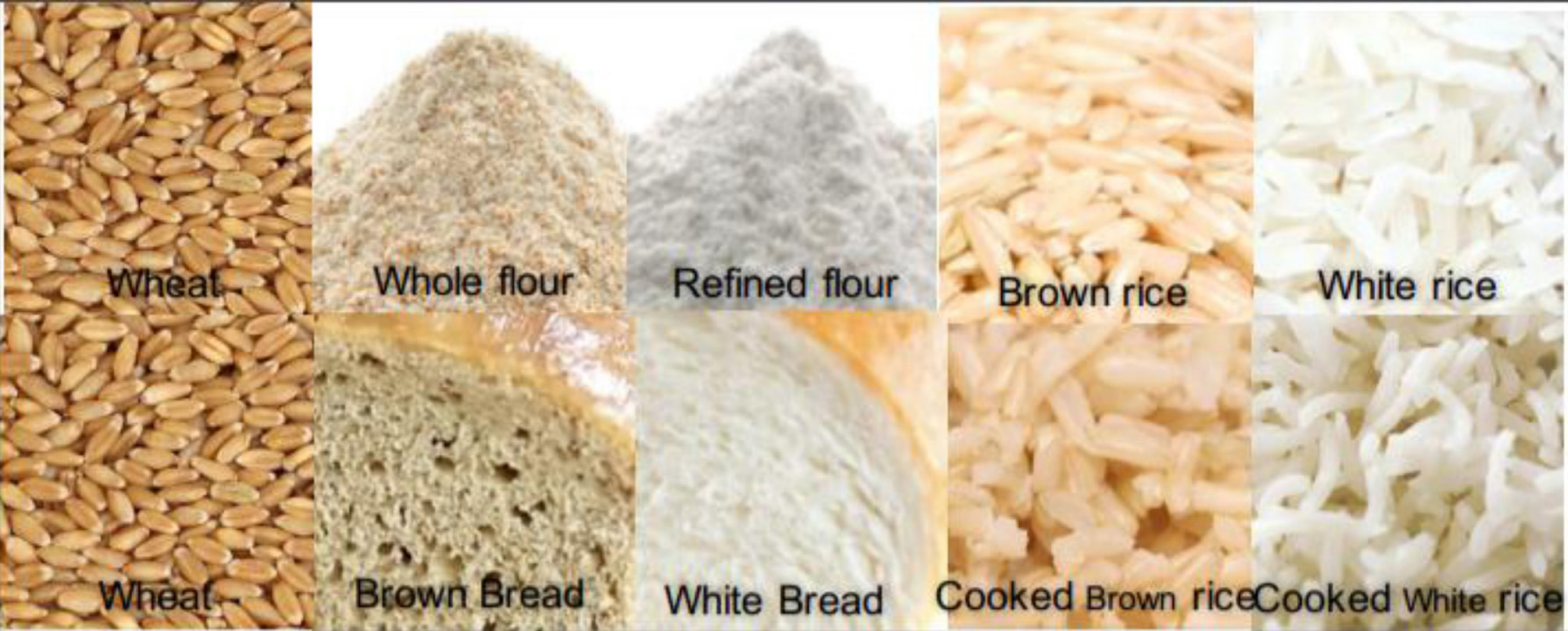									
**Vitamin content (mg per 100 g)**	**RDA (mg)**	**Bread (brown)**	**% RDA**	**Bread (white)**	**% RDA**	**Rice (brown)**	**% RDA**	**Rice (white)**	**% RDA**
B1	1.2	0.21^b^	18	0.1^b^	8	0.178	15	0.02	2
B2	1.3	0.16^b^	12	0.11^b^	8	0.069	5	0.013	1
B3	16	3.8^c^	24	1.6^c^	10	2.561	16	0.4	3
B5	5	0.65	13	0.46	9	0.38	8	0.15	3
B6	1.3	0.14^b^	11	0.017^b^	1	0.123	9	0.093	7
B9	0.4	0.04^a^	10	0.025^a^	6	0.009	2	0.003	1
E	15	2.66	18	0.38	3	0.17	1	0.04	0
K	0.12	0.008	7	0.008	6	0.0002	0	0	0

*Adapted from USDA food data central;*

a *(7);*

b *(5)*.

### Vitamin A

Vitamin A can be obtained from two sources: animal tissue (retinol) and plant tissue (provitamin A carotenoids). Retinol, retinal, retinoic acid, and retinyl esters are different forms of retinoids, whereas α-carotene, β-carotene, and β-cryptoxanthin are different forms of provitamin A carotenoids. Provitamin A carotenoids can be converted into vitamin A during digestion in the human body. Other carotenoids, such as lycopene, lutein, and zeaxanthin cannot be converted to vitamin A; however, their consumption brings associated health benefits (22). The major carotenoids in cereal grains are β-carotene, β-cryptoxanthin, lutein, and zeaxanthin (23). The carotenoid content of rice, white wheat, and white corn is negligible. The conventional yellow corn contains <2 μg/g of provitamin A carotenoids and up to 20 μg/g of total carotenoids, biofortified corn contains 6–8 μg/g provitamin A carotenoids and up to 66 μg/g of total carotenoids, and yellow durum wheat contains 3 μg/g total carotenoids (23). The distribution of nutrients within different cereal grains is not uniform. Carotenoids are found mainly in the corneous endosperm (74–86%), followed by the endosperm (9–23%), germ (2–4%), and pericarp (1%) (24). Thus, milling/degerming of grains should increase the carotenoid content of flour. Degermed cornmeal contained around 20% higher carotenoids (25); however, the milling of durum wheat resulted in a loss of approximately 8% (23) and the milling of corn resulted in a loss of between 8 and 13% due to lipoxygenase activity (25). The average retention of carotenoids following cooking has been reported to be ~62% for yellow maize and wheat-based products (25).

### B Vitamins

B vitamins are found mainly in the seed coat and germ. In terms of crops, these are restricted to the scutellum and aleurone layer in wheat; the germ and aleurone layer in rice; and the germ and hull in corn (26, 27). Cereals are a source of almost all B vitamins, except B12 (Table 1). Although the vitamin content of whole cereals is high, their values are significantly reduced (50–90%) after milling (Table 1). The different B vitamin contents of cereals vary and are dependent on genotype, the environment of cultivation, harvesting time, storage method, and processing method. The reporting of data on cereal vitamin content is complicated by the application of different estimation methods, lab-to-lab variations, and the use of different cultivars or enrichment methods. Vitamin losses following cooking are also significant; a comparison of cooked whole and milled cereals (wheat and rice) indicated that whole grain products are superior to their milled counterparts in terms of vitamin content (Table 2). Between 10 and 24% of the RDA for different B vitamins can be provided by 100 g of whole wheat bread, while for white bread this value varies between 1 and 10%; similarly, for cooked brown rice, a value between 0 and 16% is provided compared with cooked white rice between 0 and 7%.

### Vitamin E

Of the eight vitamin E compounds, plants contain two types of tocochromanols (tocols): tocopherols and tocotrienols (28). The majority of the tocols are found in the oil-rich germ layer (26), and as the germ layer in corn is larger, its concentration in corn is also higher. Degerming and milling remove between 90 and 95% of the vitamin E content. Similar to B vitamins, whole wheat bread is a good source of vitamin E (18% RDA/100 g), while in cooked white rice these are lost (0% RDA).

### Vitamin K

Vitamin K1 (phylloquinone), K2 (menaquinones), and K3 (menadiones) are three basic types of vitamin K (29). Cereals are not a good source of vitamin K, as shown in Table 1. Vitamin K losses following milling vary from 80 to 85% and cooking further reduces its content in the final food product. Whole wheat bread provides 7% RDA of vitamin K, while it is completely lost in cooked white rice (0% RDA) (Table 2).

## Vitamins in Cereal Grass Juices

Cereal grasses of wheat, alfalfa, barley, oat, or a combination of these grasses are used as herbal medicines due to their therapeutic and nutritional properties. According to literature reports, cereal grasses contain a variety of nutrients, including vitamins, proteins, minerals, and carbohydrates (30). Among all the cereal grasses, wheatgrass is the most popular and readily available in the market as a health food supplement in the form of juice, dried powder, tablets, and capsules. Wheat seedlings collected after 6–10 days of germination are called wheatgrass. Wheatgrass is a good source of vitamin A, C, E, K, and B complexes (31). Prior reports indicated that there is a variation in the vitamin content of wheatgrass juice and powder that is dependent on the source, harvest time, growth method, and production method. The most efficient method to prevent micronutrient loss from wheatgrass is to produce a freeze-dried powder; however, this is an expensive method. A forced-air shade drying method was found to be a cost-effective method to dry fresh wheatgrass that offered lower drying times and higher chlorophyll contents (32). The recommended serving amount per day is 30 ml (one fluid ounce) for juice and 3.5 g (one teaspoon) for powder (6). Table 3 shows an analysis of the vitamin content of dehydrated wheatgrass powder and the RDA, based on a 2000-calorie diet (6, 33). The use of a powdered form of wheatgrass has proven to be cost-effective as the vital nutrients are retained and the shelf life is improved, in comparison to wheatgrass juice. Wheatgrass juice is not harmful, but it can cause an allergic reaction in some individuals; therefore, for most people, wheatgrass can be consumed as a part of their daily dietary intake. Apples are generally regarded as a healthy fruit with high contents of vitamin A (3.7% RDA), B9 (9.3% RDA), and C (6.3% RDA) per serving. A serving of carrot only provides a rich source of vitamin A (29% RDA), whereas wheatgrass provides a wide range of nutrients. Per serving, wheatgrass contains high amounts of B2 (46% RDA), B9 (10% RDA), and C (8.3% RDA). Thus, wheatgrass can be classed as a nutraceutical category, provided more studies support the current literature.

**Table 3:** Vitamin content present in wheatgrass dehydrated powder.

**Vitamin**	**Content (mg/100 g)**	**Content (mg/3.5 g)**	**RDA (mg)**	**%RDA serving**	
A	0.3	0.01	0.9	1	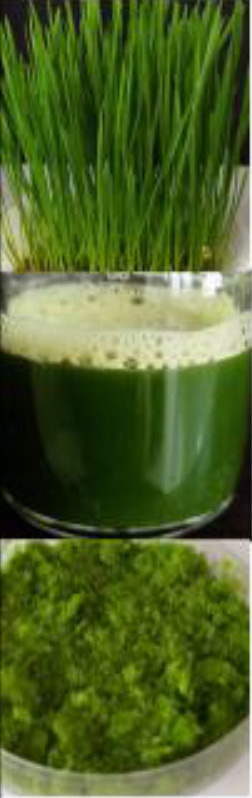
B1	0.3	0.01	1.2	0.8	
B2	17	0.6	1.3	46	
B3	8.3	0.3	16	1.8	
B5	0.7	0.02	5	0.4	
B6	1.4	0.04	1.3	3.8	
B9	1.1	0.03	0.4	10	
C	214.5	7.5	90	8.3	
E	9.1	0.3	15	2	

*RDA, Recommended daily allowance for adult male 19–50 years based on a 2,000 calorie diet*.

### Vitamins A and E

A 3.5 g serving of the whole leaf dehydrated wheatgrass powder contains 0.01 mg (1% RDA) of vitamin A compared to the 0.26 mg (29% RDA) in 50 g of raw carrot and 0.03 mg (3.7% RDA) in apple (125 g of apple per serving) (6, 33). It also contains 0.3 mg (2% RDA) of vitamin E compared to 20.3 mg (135% RDA) in 7.29 g of wheat germ oil and 0.2 mg (1.5% RDA) in apple (6, 33).

### B Vitamins

A 3.5 g serving of wheatgrass powder contains 0.01 mg (0.8% RDA) of vitamin B1 compared to 0.24 mg (20% RDA) in 100 g of boiled black beans and 0.02 mg (1.8% RDA) in apple. There is 0.6 mg (46% RDA) of vitamin B2 in wheatgrass powder compared to 0.3 mg (23% RDA) in 28.3 g of dry roasted almonds and 0.03 mg (2.5% RDA) in apple. It also contains 0.3 mg (1.8% RDA) of vitamin B3 compared to 4.2 mg (26% RDA) in 28.3 g of dry roasted peanuts and 0.2 mg (0.7% RDA) in apple. Wheatgrass has 0.02 mg (0.4% RDA) of vitamin B5 compared to 0.8 mg (16% RDA) in 40 g fried white mushrooms and 0.07 mg (1.5% RDA) in apple. It contains 0.05 mg (3.8% RDA) of vitamin B6 compared to 0.4 mg (30% RDA) in 118 g of banana and 0.05 mg (4% RDA) in apple. Wheatgrass contains 0.04 mg (10% RDA) of vitamin B9 compared to 0.05 mg (12% RDA) in 90 g of cooked broccoli and 0.03 mg (9.3% RDA) in apple (6, 33).

### Vitamin C

The concentration of vitamin C in wheatgrass powder is 7.5 mg (8.3% RDA) compared to 70 mg (77% RDA) in 154 g of orange and 5.7 mg (6.3% RDA) in apple (6, 33).

## Cereal Fortification

Food fortification is a method of deliberately increasing the content of essential micronutrients in a portion of food. Many plants, bacteria, and yeast synthesize vitamins that mammals are not able to. Food-to-food fortification of staple cereal products provides promise in combatting vitamin A, C, and D deficiencies. The use of microorganisms to deliver vitamins is significant in the production of functional foods with enriched vitamins. Food fortification of staple cereal products with vitamins B2, B9, B12, and K using microorganisms (bacteria and yeast) and direct fortification with vitamins A, B1, B2, B3, B6, B9, B12, D, and E (synthetic sources) can aid with the prevention of vitamin deficiencies (Table 4).

**Table 4:** Fortification of cereals to address deficiency disorders.

**Vitamins**	**Natural source used**	**Artificial source used**	**References**
A	*Adansonia digitate*		(34)
	*Moringa oleifera*		(35)
	Guava skin		(36)
B1		Addition of 6.4 mg/kg of B1 to cereal flour	(37)
Vitamin B2	Pap, akamu, ogi, koko, fermented maize, millet, sorghum, soyabean	Addition of 3.9 mg/kg B2 to cereal flour	(27, 37–39)
B3		Addition of 52.9 mg/Kg B3 to cereal flour	(37)
B6		Daily dietary intake is 4.18 mg/kg	(40)
B9	Addition of Lactobacillus to cereals	Addition of 1.5 mg/Kg of B9 to cereal flour	(37, 41)
B12	Fermented Wheat bran with *Propionibaterium freudenreichii* and *Lactobacillus brevis*		(42)
C	Fermented *Afzelia africana* legume flour		(43, 44)
D		Direct addition of 0.024 mg/kg of Vitamin D to foods	(45)
	Whole grain or pearled barley flour		(46)
K	Fermented soyabean		(47)

### Fat Soluble Vitamins

Carrots, mangoes, moringa seeds, and guava skin have been used to fortify foods with vitamin A. For example, the fortification of pearl millet porridge with carrots, mango, and African baobab fruit (48, 49), the fortification of bread with moringa seed powder (34, 50), and the fortification of cake with guava skin (35) have been reported. The direct fortification of foods with vitamin D at a level of 0.024 mg/kg is an effective strategy to increase vitamin D levels in humans (51, 52). To fulfill the demand for vitamin E for consumption, pita bread has been fortified with either whole grain or pearl barley flour (36). To meet the nutritional requirement for the consumption of vitamin K, a soybean product fermented with *Bacillus subtilis*, natto, has been reported (45).

### B Vitamins

The FDA mandates that to increase the nutrient quality wheat flour should be fortified with 6.4 mg/kg of vitamin B1 (37) and many other countries have also made this mandatory. The FDA has recommended vitamin B2 fortification at a rate of 3.9 mg/kg (37). To support the nutritional demand for vitamin B2, fermented cereal products, such as Pap, Akamu, Ogi, or koko fortified with *Lactobacillus plantarum* have been reported (46, 47, 53). The FDA has recommended vitamin B3 flour fortification at a rate of 52.9 mg/kg (37) and many countries have also made this mandatory. In the United States, the daily average dietary intake of vitamin B6 from food is approximately 4.2 mg/kg (38). To justify mandatory vitamin B6 fortification, rigorous studies are required to analyze the exact rate of deficiency. Currently, there are no vitamin B6 fortification strategies in use for any foods. However, it is commonly added to breakfast cereals to increase the levels of pyridoxal phosphate in plasma (39). The FDA has recommended vitamin B9 fortification at a rate of 1.5 mg/kg (37). An example is the use of yeast to increase the vitamin B9 content in cereals (40). Non-sterile wheat bran co-fermented with two types of bacteria, *Propionibacterium freudenreichii* and *Lactobacillus brevis*, has been used to increase the vitamin B12 content (54). In addition, bread co-fortified with vitamins B12 and B9 have been reported (41).

### Vitamin C

A variety of different fortification strategies have been used to fortify cereals with vitamin C. The fortification of cookies using blended brown rice and fermented legume flour (*Afzelia africana*) has been reported (42, 55).

The use of food fortification is increasing exponentially with time. It may not completely eliminate dietary inadequacies, but it does help increase the proportion of adults who meet their daily estimated average requirement (43). Food enrichment and food fortification programs in the United States have contributed to increased nutrient intake in the general population and the eradication of several diseases linked to vitamin or mineral deficiency. For example, folic acid fortification has significantly decreased the incidence of neural tube defects. Based on their analysis of NHANES 2003–2006 data, Fulgoni et al. (43) reported that the food fortification and enrichment programs were key contributors of nutrient sufficiency and, along with nutrient supplements helped decrease the percentage of the population that consumed less than the estimated average requirement of key nutrients. The RDAs were established to meet the nutritional needs of the majority of the healthy population (56).

## Biofortification Methods to Increase Cereal Vitamin Content

Biofortification offers a long-term solution to improve the nutritional quality of food crops and prevent micronutrient deficiencies. It can be achieved *via* agronomic practices, conventional plant breeding, genetic engineering, and modern biotechnological processes (44). Several vitamins including A, B1, B2, B3, B6, B9, B12, C, and E are used in the direct biofortification of crops (Table 5). As the daily requirement for vitamins B5, B7, and K is easily fulfilled from most natural dietary sources, biofortification using these vitamins is rare.

**Table 5:** Biofortification of cereals to increase their vitamin content (mg/100 g).

**Vitamin**	**Crop**	**Gene**	**Original**	**Enhanced**	**Fold change**	**%RDA Original**	**%RDA Enhanced**	**References**
A				**BREEDING TECHNIQUE**				
	Maize (β-carotene)	Chx/reduction	0.23	1.8	8	0.1	**100**	(57, 58)
	Maize (β-carotene)	Chx/reduction	0.14	1.3	9.3	0.08	**72**	(59, 60)
				**GENETIC MODIFICATION**				
	Maize (β-carotene)	*PSY, CrtI*	0.035	5.9	169	0.02	**328**	(61, 62)
	Wheat (β-carotene)	*psy1, Chx* (reduction)	0.016	0.5	31	0.01	28	(63, 64)
	Wheat (Total carotenoid)	*psy1, CrtI*	0.046	0.5	10.8	0.03	28	(65, 66)
	Rice (β-carotene)	*psy1, CrtI*	0.16	3.7	23	0.09	**206**	(67, 68)
	Sorghum (β-carotene)	HGGT	0.05	1.23	24.6	0.03	**68**	(57, 69)
B1				**GENETIC MODIFICATION**				
	Rice	*THIC, THID*	0.34	1.7	5	0.14	**71**	(63, 70)
B3 (Tryptophan%)				**BREEDING TECHNIQUE**				
	Maize	*o2*	0.004	0.007	1.7	NA	NA	(59, 71)
	Maize	*o2, o16*	0.004	0.012	3	NA	NA	(65, 72)
B6				**GENETIC MODIFICATION**				
	Rice	PDX1, PDX2	0.1	0.32	3.1	0.04	12	(73, 74)
B9	Rice	*GTPCHI8, ADCS*	0.017	1.7	100	0.02	**213**	(73, 75)
		*GTPCHI8, ADCS, FPG*	0.017	2.53	150	0.02	**316**	(76, 77)
	Wheat	*Gm8gGCH1, LeADCS*	0.028	0.06	2.3	0.035	7.5	(78, 79)
	Corn	*GTPCH1, ADCS*	0.093	0.2	2	0.12	25	(61, 62)
C				**GENETIC MODIFICATION**				
	Corn	DHAR	1.75	10.7	6	0.01	5.9	(61, 62)
E (α-tocopherol)				**BREEDING TECHNIQUE**				
	Corn	*VTE4*-mutation	0.476	3	6.3	0.02	10	(4, 69)
		*VTE4*-transfer	0.64	4.2	6.5	0.02	14	(80)
				**GENETIC MODIFICATION**				
	Corn	*HPPD, MPBQ-MT*	0.29	0.9	3	0.01	3	(62, 81)
		*GmTMT2a*	0.1	4.5	4.5	0.003	15	(82, 83)

*%RDA has been calculated after taking into account 50% processing losses*.

*PSY, phytoene synthase; Chx, β-carotene hydroxylase 1; CrtI, carotene desaturase; HGGT, Homogentisate geranyl geranyl tranferase; THIC, 4-amino-2-methyl-5-hydroxymethylpyrimidine monophosphate synthase; THID, HMP-P kinase; PDX, pyridoxal 5′-phosphate synthase; GTPCHI, triphoshpate cyclohydrolase I; ADCS, aminodeoxychorismate synthase; FPG, folate binding protein; DHAR, dehydroascorbate reductase; TMT, γ-tocopherol methyl transferase; HPPD, 4-hydoxyphenyl-pruvate dioxygenase; MPBQ-MT, 2-methyl-6-phytyl-1,4-benzoquinolmethyltransferase. The bold values are showing drastic increase in %RDA of vitamins*.

### Vitamin A

Various strategies have been used to increase the provitamin A content of cereals.

#### Breeding Technique

The loci lcyE (lycopene epsilon cyclase) and Chx (β-carotene hydroxylase 1) have been identified as key targets for provitamin A biofortification and molecular markers for these loci are being employed to improve maize crops as it has high genetic diversity for this trait (84). This genetic variability has been used for the development of biofortified maize varieties or hybrids. International Maize and Wheat Improvement Center (CIMMYT) has been extensively working on the development and dissemination of these varieties in Africa. For example, several maize varieties have been developed and released in countries such as Zambia, Nigeria, Ghana, Malawi, Tanzania, and Zimbabwe (44, 84, 85). Similarly, sorghum is being explored for provitamin A biofortification (86). Biofortified orange maize is as efficacious as vitamin A supplementation in the experiment carried out in Zambia (18).

Techniques such as quantitative trait locus (QTL) mapping and genome-wide association studies (GWAS) have been developed to identify key targets, e.g., the ZEP gene (zeaxanthin epoxidase) in sorghum (87) and major loci in durum wheat (88). The targeting-induced local lesions in genomes (TILLING) approach, has been utilized to create durum wheat genetic lines that contain high levels of provitamin A carotenoids by silencing the lcyE-A and lcyE-B genes (89). Further, a pyramiding of these lines into a single line has been shown to cause further increase in the provitamin A carotenoid content in crops (90).

#### Metabolic Engineering

Daffodil phytoene synthase (PSY) and bacterial carotene desaturase (CrtI) have been exploited to improve the β-carotene levels in golden rice by a factor of four (91). The replacement of daffodil PSY with maize PSY resulted in 23-fold improvement in β-carotene levels in golden rice variety 2 (68). A combination of PSY and CrtI has been reported to increase carotenoid levels 169-fold in maize and 10.8-fold in wheat (Table 5), whereas a combination of PSY and Chx increased the levels 31-fold in wheat. Over-expression of homogentisate geranylgeranyltransferase (HGGT) has been reported to increase β-carotene levels 24.6-fold (Table 5). Combining the over-expression of bacterial PSY and the silencing of Chx, *via* RNA interference has been shown to increase β-carotene levels 30-fold in wheat endosperm (64).

### Vitamin B1

#### Agronomic Approach

Fodder plants irrigated with sewage water contain a higher level of vitamin B1 compared to those irrigated with clean water. For wheat, the application of cow dung or sugarcane bagasse in the fields has been shown to improve the vitamin B1 content of the plants (92).

#### Breeding Technique

Genetic diversity has been observed in the vitamin B1 content in wheat and cultivars with higher contents, e.g., Lumai 15, Jimai 19, Aifeng 3, and Bima 2 (93). The application of GWAS and QTL mapping identified that multiple QTLs contribute to the vitamin B1 content of common wheat (67). Single nucleotide polymorphism (SNP) markers, such as IWB43809 (6AS, 0Cm) and IWB69903 (6AS, 13cM), have been reported to be associated with the vitamin B1 content of wheat (93).

#### Genetic Modification

The over-expression of 4-amino-2-methyl-5-hydroxymethylpyrimidine monophosphate (HMP-P) synthase (THIC) and HMP-P kinase (THID) has been shown to lead to a 2.4-fold increase in the thiamin level in rice leaves and up to a 5-fold increase in unpolished rice seeds (63).

### Vitamin B2

Various cultivars with higher levels of vitamin B2, such as Lankao2, Mantol, and Funo, have been identified. Through the application of GWAS, SNP markers such as IWB23595 (1DS, 68cM), IWA8005 (5BL, 49cM), and IWB65016 (7BL, 159Cm) are associated with the vitamin B2 content of wheat and maybe the subject of further research into increasing the vitamin content of wheat (93).

### Vitamin B3

The natural opaque-2 (o2) mutation that is being exploited as a quality protein maize (QPM) leads to the synthesis of increased levels of the essential amino acids lysine and tryptophan (94). It has also been shown to increase the level of vitamin B3 equivalents due to the availability of more tryptophan and the subsequent conversion to nicotinamide (95). Furthermore, by combining the o2 and o16 mutants, an increase in lysine and tryptophan of between 40 and 80% has been observed in comparison with the o2 mutant alone (96). Molecular markers for o2 are being employed in the improvement of maize (70, 97). CIMMYT has been extensively working in different countries for the development and dissemination of QPM varieties (84). Several QPM maize varieties have been released in Africa, Latin America, and Asia (97).

### Vitamin B6

Germplasm screening of crops has revealed limited variations (<2-fold) in the vitamin B6 composition of wheat (98). Constitutive expression of two *Arabidopsis* pyridoxal 5′-phosphate synthase genes (PDX1 and PDX2) has resulted in a considerable increase in the level of vitamin B6 in rice leaves (up to 28.3-fold), roots (up to 12-fold), and seeds (up to 3.1-fold) (74).

### Vitamin B9

#### Agronomic Approach

During sweet corn seed germination, the folate content and the expression of aminodeoxychorismate synthase (ADCS), dihydrofolate reductase (DHFR), and guanosine triphosphate cyclohydrolase (GTPCH) were increased in light conditions in comparison to dark conditions (99).

#### Breeding Technique

Genetic diversity has been observed in the vitamin B9 content in rice and maize and QTLs have been identified for increasing B9 content in these crops. Three major QTLs: qQTF-3-1, qQTF-32, and qQTF-3-3, were identified in milled rice (chromosome 3) (100) and two major QTLs, q5-F-THF-a and q5-F-THF-b, were identified in maize (chromosome 5) (101).

#### Genetic Modification

Enhancement in the activity of GTPCHI and ADCS has been targeted to increase the level of vitamin B9 (95) and has been implemented in rice (73) and maize (62). The ectopic expression of GTPCH1 and ADCS led to a 100-fold enhancement in vitamin B9 levels in rice (73). Furthermore, the introduction of additional folate-binding protein, with both increased GTPCHI and ADCS led to a 150-fold increase in folate compared with wild-type rice (77). The co-expression of glycine max, Gm8gGCH1 and GmADCS, genes resulted in a 2.3-fold increase in folate content in wheat and a 4.2-fold increase in corn (75).

### Vitamin B12

Barley treated with pure vitamin B12 or cow dung has been shown to increase the level of vitamin B12 in the seeds (61). However, no studies have reported the biofortification of crops with vitamin B12 due to the presence of an exclusive biosynthetic pathway in bacteria and some archaea (95).

### Vitamin C

#### Agronomic Approach

An increase in the vitamin C content of wheat leaves and kernels has been induced *via* the exogenous supply of l-galactono-1,4-γ-lactone (GaL) (76). During germination, corn seeds exposed to a high amount of light exhibited increased vitamin C content and higher expression of the VTC2 (GDP-l-galactose phosphorylase) and l-galactono-1,4-lactone dehydrogenase (GLDH) genes (99). The production of reactive oxygen species (ROS) in plants under high light-exposure conditions explains the increased level of expression of dehydroascorbate reductase (DHAR) during light germination (78).

#### Breeding Technique

A QTL mapping technique has been employed to identify the multiple QTLs contributing to the vitamin C content in edible crops (76). Tomatoes have been investigated as a model crop (102). In comparison with the parental lines, maize heterotic F1 hybrids (B73^*^Mo17) have a higher vitamin C biosynthesis capability (76).

#### Genetic Modification

The over-expression of GDP-l-galactose phosphorylase (GGP) has been proved to be a useful strategy for biofortification using vitamin C (103). The variety of rice, IR64, has been engineered to express the kiwifruit GGP gene, leading to a 2.5-fold increase in foliar ascorbate levels (104). The over-expression of DHAR, along with the use of a specific endosperm barley d-hordein promoter resulted in a 6-fold increase in vitamin C levels in corn (62).

### Vitamin E

#### Agronomic Approach

Under a light environment, it has been shown that sweet corn sprouts had higher vitamin E levels; the amount of δ-tocotrienol increased from 18 to 26% and from 16 to 20% in the later stages of sweet corn development under light and dark conditions, respectively (99). A significant increase in α-tocopherol under light conditions has been observed in sweet corn seeds during the second and the third stages of germination (105).

#### Breeding Technique

Germplasm screening has identified high α-tocopherol cultivars in rice (106). QTL studies identified VTE4 (TMT, γ-tocopherol methyltransferase) as the major genetic locus in maize kernels for the efficient conversion of γ-tocopherol to α-tocopherol. Insertion or deletions (InDel1, InDel4, InDel7, and InDel118) within the VTE4 gene were found to significantly affect the level of α-tocopherol. Introgression of the favorable allele of TMT using marker-assisted selection into sweet corn inbreds resulted in enhancement of α-tocopherol (80, 97).

#### Genetic Modification

The over-expression of γ-TMT is associated with enhanced vitamin E activity in different crops (107). The synchronous expression of 4-hydoxyphenyl-pruvate dioxygenase (HPPD) and 2-methyl-6-phytyl-1,4-benzoquinolmethyltransferase (MPBQ-MT) tripled the tocopherol level in corn kernels (108).

Biofortification has emerged as a viable and effective route for delivering nutrient-rich crops to combat hidden hunger with minimal recurrent investments after dissemination (109). Consuming biofortified crops can address micronutrient deficiency by increasing the daily adequacy of micronutrient intake among individuals. The key advantages include the long-term cost-effectiveness in comparison to fortification and supplementation approaches, and also, they are a feasible means of reaching rural populations who may have limited access to diverse diets or other micronutrient interventions (82). Provitamin A-biofortified maize, an efficacious source of vitamin A has been demonstrated to improve total body vitamin A stores and to significantly improve visual function in marginally vitamin A-deficient children (18, 110). Provitamin A-rich rice, termed Golden Rice, is a good example of a product withgreat potential (81). QPM developed by the CIMMYT scientists has received the 2000 World Food Prize. QPM with high lysine and tryptophan content is more nutritious than conventional maize, and village-level evidence suggests that consuming a high-maize diet can improve the health of children (111). The vitamin B3 value could not be calculated as it is produced by the body from QPM; however, its efficiency in reducing the symptoms associated with the vitamin B deficiency disease, pellagra, has been documented (112). Over 20 million people worldwide are currently consuming biofortified crops (82). But there is a lag before any health benefits of a biofortified crop are realized due to the time needed to implement new crop strategies (113).

## Effect of Cooking on the Vitamin Content of Cereals

Cooking can have a significant effect on the content of vitamins in cereals, and lead to imprecise estimation of nutrient intake. Therefore, it is essential to ascertain nutritional information related to the retention of vitamins in cereals and different cooking methods. The highest retention of vitamins in cereals is observed when they are boiled and the lowest following pressure cooking (Table 6).

**Table 6:** Effect of different cooking methods on vitamins content in cereals.

**Vitamins**	**Cereal crop**	**% Retention**	**% Cooking loss/gain**	**Cooking method**	**References**
A	Maize (Y)	64	−36	Nixtamalized snack	(114)
	Maize (Y)	122.4	23	Boiling	(83)
		24.8	−75	Porridge	
		80	−20	Tortilla	
	Maize (Y)	20	−80	Boiling (G)	(115)
		34	−66	Boiling (Flo)	
		90	−10	Steaming (Ba)	
		95	−5	Steaming (S)	
		205	105	Steaming (D)	
		69	−31	Popping (WO)	
		78	−22	Popping (O)	
		78	−22	Frying (Fl)	
	Maize (O)	68	−32	Porridge	(116)
		103	3	Samp	
		98	−2	Phutu	
	Maize (W, F)	40	−60	Porridge (F)	(117)
	Maize (B)	112	12	Porridge	(89)
B1	Rice	89.5	−11	Boiling	(118)
		52.68	−29	Parboiling	
		49.66			
		54.0370.9			
		67.9	−32	Pressure	
		54.4	−46	Microwave	
		51	−49	Microwave	(87)
		32.5	−68	Pressure	
	Rice (F)	74	−26	Boiling	(79)
		65.41	−34.6	Microwave	(119)
			−47.3	Boiling	
			−50.3	Stir frying + Boiling	
			−46.0	Boiling (LS)	
	Wheat (F pasta)	85.5	−15	Boiling	(52)
B2	Rice	60	−40	Microwave	(87)
		51	−49	Pressure	
	Rice (F)	71	−29	Boiling	(79)
	Wheat (F pasta)	82.77	−17.2	Boiling	(52)
B3	Rice (F)	82.6	−17.4	Boiling	(79)
	Wheat (F pasta)	12	−88	Boiling	(52)
B5	Rice (F)	77	−23	Boiling	(79)
B6	Rice (F)	77	−23	Boiling	(79)
	Rice	93	−7	Boiling	(118)
		87	−13	Parboiling	
		63	−37	Pressure	
		45	−55	Microwave	
B9	Wheat	80	−20	Cake	(120)
		87	−13	Bread	
		57	−43	Cream sauce	
	Wheat	84	−16	Steam buns	(121)
		89	−11	Breads	
	Maize	99	−1	Cake	(120)
		97	−3	Couscous	
	Rice	91.1	−9	Boiling	(118)
		87.9	−12	Parboiling	
		55.2	−45	Microwave	
		79.8	−20	Pressure	
	Rice (F)	83.63	−16	Microwave	(119)
		84.21	−16	Boiling	
		96.11	−4	Stir frying + Boiling	
		75.69	−24	Boiling (LS)	
B12	Rice (F)	54	−46	Boiling	(79)
C	Rice	44.5	−56	Boiling	(118)
		49.8	−50	Parboiling	
		32.4	−68	Microwave	
		17.5	−83	Pressure	
E	Barley(Bulgar)	88	−12	Steam	(122)
		82	−18	Microwave	
		55	−45	Ovendried	
	Rice (BR) (Noodles)	85.5	−14.5	Boiling	(123)
	Rice (C)	114	14	Boiling	(124)
	Rice (BR)	10	−90	Parboiling + storage + boiling	(56)

*G, Grits; S, Sweetcorn; D, Dent corn; O, Oil; WO, Without Oil; Fl, Flakes; Flo, Flour; B, Biofortified; Ba, Baby corn; F, Fortified; O, Orange; W, White; Y, Yellow; BR, Brown; F, Fermented; C, Colored; LS, Large scale*.

### Vitamin A

In maize, carotenoids are mainly found in the endosperm with lower amounts in the bran and germ. An increase in the total carotenoid concentration has been reported after milling refined flour (125). Cooking has been shown to increase the level of carotenoids in steamed dent corn, boiled kernels, and coarsely ground maize samp. The maximum vitamin A losses were observed in finely ground corn porridge, grits (nixtamalized whole cornmeal), cornflour, and nixtamalized snacks, whereas medium losses were observed following the popping of corn, frying tortillas, and flaking. Boiling and steaming have been shown to cause lower reductions in coarsely ground phutu, porridge, baby corn, and sweet corn (83, 85, 116, 125–127). The cooking temperature is an important determinant of cooking losses in carotenoids (125, 127).

### B Vitamins

Vitamin B1 losses following cooking are dependent on the method of cooking with the lowest observed losses during boiling (72, 114) and the highest in the case of pressure cooking (115, 117). The rate of vitamin B1 loss at 121°C is faster than that at 99°C or below, indicating that temperature is one of the most important factors in determining cooking losses. Similarly, vitamin B2 loss is low following boiling and higher following microwave and pressure cooking (Table 6). In pasta, the losses of vitamin B3 have been reported to be higher than B1 and B2. Vitamin B3 can leach into the cooking water; therefore, pasta products should not be cooked in excess water, and the cooking water should not be discarded (72). Following the cooking of rice, the vitamin losses (B1, B2, B3, B5, and B6) were lower for the rice fortified by soaking than for the rice fortified using a spraying method (114). Similar to other vitamins, lower vitamin B9 losses have been observed after boiling and stir-frying than those following microwave and pressure cooking (115, 117) (Table 6). The vitamin B9 losses in samples cooked using a dry-heat method (wheat flour cake, bread loaf, couscous, and corn cake) were lower than those cooked using a moist-heat method (white cream sauce) (79, 119) due to leaching into the water. The losses of vitamin B12 after boiling are intermediate (114) (Table 6).

### Vitamin C

Ascorbic acid (vitamin C) is sensitive to heat and oxidation. In rice, the loss of ascorbic acid following cooking is dependent on the variety of rice and the method used (115). Pressure cooking methods cause higher losses of vitamin C, followed by microwave cooking, conventional cooking, and finally parboiling (83–56%).

### Vitamin E

For bulgur prepared from superheated steam-dried barley grains (110°C), the vitamin E cooking losses observed were the lowest, followed by the microwave-dried grains and then the oven-dried grains, which indicates the key role played by temperature. The concentration and retention of vitamin E decreased with an increasing treatment temperature (118). Taken together, parboiling, storage, and recooking as well as extrusion cooking can cause a loss of up to 90% of the total tocol content of rice (120, 128). The loss of vitamin E after boiling is low, instead, an increase in the content has been reported in some cases, which is due to improved extraction after boiling (121, 122) (Table 6).

## Vitamin Bioaccessibility

Bioaccessibility is the fraction released from the food matrix that is available for intestinal absorption following enzymatic hydrolysis under acidic conditions. Bioaccessibility is affected by many factors, such as food type, vitamin type, amount of dietary fiber, presence of inhibitors, matrix effects, post-harvest storage, and packaging methods (2, 129). The average bioaccessibilities of vitamins B1, B2, B3, B6, B9, C, and E in cereal-based baby foods were found to be 81, 79, 45, 60, 52, 27, and 99%, respectively (123, 124).

Both cooking losses and bioaccessibility are essential to estimate the health effects of vitamins in cereal foods. The cooking losses and bioaccessibility of carotenoids in maize-based products were ~23 and 8% for boiled kernels, 20 and 20% for tortillas, and 75 and 50% for porridge, respectively (83). Considering only cooking losses, the order would be boiled kernels, tortillas, and then porridge. However, if we take both cooking losses and bioaccessibility into account, then the order would be tortillas, porridge, and boiled kernels. Bioaccessibility is also influenced by the food matrix. Supplemented banana and potato has been reported to have better carotenoids bioaccessibility than white maize (130). Among the carotenoids, β-carotene has lower stability and bioavailability than β-cryptoxanthin (97, 130). Thus, estimation of bioaccessibility is incredibly important and requires more attention.

## Conclusion

Cereals are excellent sources of vitamins A, B, and E. The data reported in the literature on vitamin content in cereals are complicated due to the inter-laboratory variations, the use of different estimation methods, and the use of different cultivars. The vitamin content can be reduced following milling and cooking. Following milling, the reductions are negligible for provitamin A due to localization in the endosperm; however, they are high (50–90%) for vitamins B and E due to localization in the seed coating and embryo, respectively. Whole wheat bread is a significantly better source of vitamins (10–24% RDA of different B vitamins per 100 g) in comparison to white bread and white rice. The consumption of orange maize biofortified with provitamin A can provide 100% RDA of vitamin A per 100 g. Flours enriched with vitamins can simultaneously address several vitamin deficiencies. Vitamin losses following cooking can be reduced by lowering the cooking temperature, pressure, and cooking duration. Although wheatgrass has been documented to be rich in vitamins B2 and B9 by several publications, more detailed studies are required to clarify this. Thus, the use of fortified and biofortified cereals and their flours is preferable, as they can provide 100% RDA, even after accounting for cooking losses.

## Author Contributions

MG drafted the manuscript layout. MK helped in overall supervision during manuscript preparation. AS, SV, VT, AK, and VM collected the literature, wrote the manuscript, and prepared tables and figures. All authors contributed to the article and approved the submitted version.

## Conflict of Interest

The authors declare that the research was conducted in the absence of any commercial or financial relationships that could be construed as a potential conflict of interest.
