# Supplementary material for: Vitamins in Cereals: A Critical Review of Content, Health Effects, Processing Losses, Bioaccessibility, Fortification, and Biofortification Strategies for Their Improvement
Source: Front Nutr. 2021 Jun 16;8:586815. doi: 10.3389/fnut.2021.586815 (PMC8241910; doi:10.3389/fnut.2021.586815)
Supplement: Supplementary file 1 [file Table_1.DOC]

Supplementary table. List of vitamins, chemical name, RDA and health benefits.

| **Vitamin** | **Chemical name** | **Source** | **RDA**  **mg/day** | **Benefits** | **Reference** |
| --- | --- | --- | --- | --- | --- |
| **A** | Retinol  Carotenoid | Meats, Dairy  Vegetables Green/yellow/ orange | Children: 0.5  Men: 0.9  Women: 0.7  Pregnant: 0.77  Lactating: 1.3 | Good vision,  healthy immune system, reproduction and growth | (i) El Sohaimy 2012  (ii) USDA  (iii) Dorosty-Motlagh et al. 2016. |
| **B1** | Thiamine | Cereals (whole grain), green vegetables, potatoes, liver, pork, eggs | Children: 0.6  Men: 1.2  Women: 1.1  Pregnant: 1.4  Lactating: 1.4 | Coenzyme in a variety of critical metabolic reactions related to energy metabolism | (i) USDA  (ii) Said 2011  (iii) Kennedy 2016 |
| **B2** | Riboflavin | Dairy, leafy vegetables, legumes, liver, kidneys, yeast, mushrooms | Children: 0.6  Men: 1.3  Women: 1.1 Pregnant: 1.4  Lactating: 1.6 | Energy production, metabolism of fats, maintain healthy blood cells and boosts metabolism | (i) USDA  (ii) Said 2011  (iii) Kennedy 2016 |
| **B3** | Niacin | Meat, fish, whole grain cereal, legumes, mushrooms, nuts | Children: 8  Men: 16  Women: 14 Pregnant: 18 Lactating: 17 | Involved in metabolic reactions that maintain the redox state of the cell, and lipid lowering effects | (i) USDA  (ii) Said 2011  (iii) Kennedy 2016 |
| **B5** | Pantothenic acid | Meat, whole grain cereals, broccoli | Children: 3  Men: 5  Women: 5  Pregnant: 6 Lactating: 7 | Essential for fatty acid synthesis and other anabolic and catabolic processes | (i) USDA  (ii) Said 2011  (iii) Kennedy 2016 |
| **B6** | Pyridoxine | Meat, fish, legumes, nuts, bananas, potatoes | Children: 0.6  Men: 1.3  Women: 1.3  Pregnant: 1.9  Lactating: 2.0 | Cofactor in a number of metabolic reactions involving carbohydrate, protein and lipid metabolism | (i) USDA  (ii) Said 2011  (iii) Kennedy 2016 |
| **B7** | Biotin | Eggs, liver, pork, leafy vegetables | Children: 0.012  Men: 0.03 Women 0.03  Pregnant: 0.03  Lactating: 0.035 | Fatty acid biosynthesis, gluconeogenesis and regulating the expression of oncogenes | (i) USDA  (ii) Said 2011  (iii) Kennedy 2016 |
| **B9** | Folic acid/folate | Leafy vegetables, legumes, citrus fruits | Children: 0.2  Men: 0.4  Women: 0.4 Pregnant: 0.6 Lactating: 0.5 | Coenzymes for cellular one-carbon metabolism, for the synthesis of thymidine and purine and treatment of neural tube defects | (i) USDA  (ii) Said 2011  (iii) Kennedy 2016 |
| **B12** | Cobalamins | Meat, fish and other animal products | Children: 0.0012 Men: 0.0024 Women: 0.0024 Pregnant: 0.0026  Lactating: 0.0028 | Production of high-turnover cells, such as red blood cells. | (i) USDA  (ii) Said 2011  (iii) Kennedy 2016 |
| **C** | Ascorbic acid | Citrus fruit, strawberries, broccoli, red peppers, potatoes | Children: 25  Men: 90  Women: 75 Pregnant: 85 Lactating: 120 | Antioxidantproperty, anti-aging, cancerand heart health | (i) El Sohaimy 2012  (ii) USDA  (iii) Moser and Chun 2016  (iv) Cobley et al. 2015 |
| **D** | Ergocalciferol | Fluid milk, margarine, fatty fish and fish Oils | Children: 0.015  Men: 0.015 Women: 0.015  Pregnant: 0.015  Lactating: 0.015 | Bone health, boost immunity and treatment of multiple Sclerosis | (i) El Sohaimy 2012  (ii) USDA  (iii) Sintzel, Rametta, and Reder 2018  (iv) Van Ballegooijen et al. 2017 |
| **E** | Tocopherol | Vegetable oils, almonds, sunflower seeds, | Children: 7  Men: 15  Women: 15 Pregnant: 15 Lactating: 19 | Anti ageing, heart disease and cancer | (i) El Sohaimy 2012  (ii) USDA  (iii) Cobley et al. 2015 |
| **K** | Phylloquinone | Leafy vegetables, soy and canola oils | Children: 0.055  Men: 0.12  Women: 0.09  Pregnant:0.09  Lactating: 0.09 | Plays a role in bone health, reduce cardiovascular diseases | (i) El Sohaimy 2012  (ii) USDA  (iii) Van Ballegooijen et al. 2017  (iv) Cheung et al. 2015 |

**Refrences**

Cheung, C. L., S. Sahni, B. M. Y. Cheung, C. W. Sing, and I. C. K. Wong. 2015. Vitamin K intake and mortality in people with chronic kidney disease from NHANES III. *Clinical Nutrition* 34 (2):235–240. [doi: 10.1016/j.clnu.2014.03.011](https://doi.org/10.1016/j.clnu.2014.03.011).

Cobley, J. N., H. McHardy, J. P. Morton, M. G. Nikolaidis, and G. L. Close. 2015. Influence of vitamin C and vitamin E on redox signaling: Implications for exercise adaptations. *Free Radical Biology and Medicine* 84:65–76. [doi: 10.1016/j.freeradbiomed.2015.03.018](https://doi.org/10.1016/j.freeradbiomed.2015.03.018).

Dorosty-Motlagh A.R., N. M. Honarvar, M. Sedighiyan, and M. Abdolahi M. 2016. The molecular mechanisms of vitamin A deficiency in multiple sclerosis. *Journal of Molecular Neuroscience* 60 (1): 82-90. [doi: 10.1007/s12031-016-0781-0](https://doi.org/10.1007/s12031-016-0781-0).

El Sohaimy, S. A. 2012. Functional foods and nutraceuticals-modern approach to food science. *World Applied Sciences Journal* 20 (5):691-708.

Kennedy, D. O. 2016. B vitamins and the brain: Mechanisms, dose and efficacy—A review. *Nutrients* 8 (2):68. [doi: 10.3390/nu8020068](https://doi.org/10.3390/nu8020068).

Moser, M. A., and O. K. Chun. 2016. Vitamin C and heart health: a review based on findings from epidemiologic studies. *International journal of molecular sciences* 17 (8):1328. [doi: 10.3390/ijms17081328](https://doi.org/10.3390/ijms17081328).

Said, H. M. 2011. Intestinal absorption of water-soluble vitamins in health and disease. *Biochemical Journal* 437 (3):357-372. doi: [10.1042/BJ20110326](https://doi.org/10.1042/BJ20110326).

Sintzel, M. B., M. Rametta, and A. T. Reder. 2018. Vitamin D and multiple sclerosis: a comprehensive review. *Neurology and therapy* 7 (1):59-85. doi: [10.1007/s40120-017-0086-4](https://doi.org/10.1007/s40120-017-0086-4).

United States Department of Agriculture (USDA). 2019. USDA Food Composition Databases. Accessed on June 12, 2020. <https://fdc.nal.usda.gov/>.

Van Ballegooijen, A. J., S. Pilz, A. Tomaschitz, M. R. Grübler, and N. Verheyen. 2017. The synergistic interplay between vitamins D and K for bone and cardiovascular health: a narrative review. *International Journal of Endocrinology* 2017. doi: 10.1155/2017/7454376.
